# The Interplay among Wnt/β-catenin Family Members in Colorectal Adenomas and Surrounding Tissues

**DOI:** 10.3390/biomedicines12081730

**Published:** 2024-08-02

**Authors:** Domenica Lucia D’Antonio, Fabiana Fantini, Carmelo Moscatello, Alessio Ferrone, Stefano Scaringi, Rosa Valanzano, Ferdinando Ficari, Konstantinos Efthymakis, Matteo Neri, Gitana Maria Aceto, Maria Cristina Curia

**Affiliations:** 1 Department of Medical, Oral and Biotechnological Sciences, “Gabriele d’Annunzio” University of Chieti-Pescara, 66100 Chieti, Italy; domenica.dantonio@unich.it (D.L.D.); fanfaby@gmail.com (F.F.); carmelo.moscatello@unich.it (C.M.); alessioferrone@yahoo.it (A.F.); gitana.aceto@unich.it (G.M.A.); 2 Villa Serena Foundation for Research, Via Leonardo Petruzzi 42, 65013 Città Sant’Angelo, Italy; 3 Department of Clinical and Experimental Medicine, University of Florence, Largo Brambilla 3, 50134 Firenze, Italy; stefano.scaringi@unifi.it (S.S.); rosa.valanzano@unifi.it (R.V.); ferdinando.ficari@unifi.it (F.F.); 4 Department of Medicine and Aging Sciences, “Gabriele d’Annunzio” University of Chieti-Pescara, 66100 Chieti, Italy; efkn78@gmail.com (K.E.); mneri@unich.it (M.N.)

**Keywords:** CRA, colorectal adenoma, *APC*, *Wnt3a*, *Wnt5a*, *LEF1*, *BCL9*, polyps, early carcinogenesis, Wnt/β-catenin

## Abstract

Background: The colorectal adenoma undergoes neoplastic progression via the normal epithelium–adenoma–adenocarcinoma sequence as reported in the Vogelgram. The hazard of developing a tumor is deeply associated with the number and size of adenomas and their subtype. Adenomatous polyps are histologically categorized as follows: approximately 80–90% are tubular, 5–15% are villous, and 5–10% are tubular/villous. Given the higher risk of a malignant transformation observed in tubular/villous adenomas, patients diagnosed with adenomatous polyposis are at an improved risk of developing CRC. The Wnt/β-catenin pathway plays a key role in the onset of colorectal adenoma; in particular, intestinal cells first acquire loss-of-function mutations in the *APC* gene that induce the formation of adenomas. Methods: Wnt/β-catenin pathway *APC*, *Wnt3a*, *Wnt5a*, *LEF1*, and *BCL9* genes and protein expression analyses were conducted by qRT-PCR and western blot in 68 colonic samples (polyps and adjacent mucosa) from 41 patients, of which 17 were affected by FAP. Ten normal colonic mucosal samples were collected from 10 healthy donors. Results: In this study, both the *APC* gene and protein were less expressed in the colon tumor compared to the adjacent colonic mucosa. Conversely, the activated β-catenin was more expressed in polyps than in the adjacent mucosa. All results confirmed the literature data on carcinomas. A statistically significant correlation between *Wnt3a* and *BCL9* both in polyps and in the adjacent mucosa underlines that the canonical Wnt pathway is activated in early colon carcinogenesis and that the adjacent mucosa is already altered. Conclusion: This is the first study analyzing the difference in expression of the Wnt/β-catenin pathway in human colorectal adenomas. Understanding the progression from adenomas to colorectal carcinomas is essential for the development of new therapeutic strategies and improving clinical outcomes with the use of APC and β-catenin as biomarkers.

## 1. Introduction

The prevalence of colorectal adenomas (CRAs) increases with age, mainly in Western populations—30–40% in the people over 50 years, predominantly in men [1,2]. The annual rate of adenoma progression to colorectal cancer (CRC) is ~0.25% [3].

CRA is associated with CRC, and at least 80% of CRC undergoes neoplastic progression via the normal epithelium–adenoma–adenocarcinoma sequence as reported in the Vogelgram model [4,5]. The incidence of cancer after a negative colonoscopy is significant because adenomas may be missed during a colonoscopy, or biological changes in the tumor growth rate may occur [6]. Screening and surveillance programs can help identify precursor lesions and prevent death from CRC [7]. Thus, it is important to understand the progression from CRA to carcinomas to facilitate the development of novel treatment strategies and improve clinical outcomes.

The malignancy of adenomas is highly correlated with the occurrence of colon cancer, depending on the subtype [8]. Furthermore, the risk of developing cancer is closely linked to the number and dimensions of previously identified polyps [9]. Developing multiple colonic polyps with malignant potential increases the lifetime risk of developing colorectal cancer (CRC). There are at least three types of polyps based on the histology and molecular pathway: adenomatous, serrated, or hyperplastic (non-neoplastic) [10,11,12]. The first type is characterized by the adenomatous histotype, while both sessile/traditional serrated adenomas and hyperplastic polyps have a serrated histotype [13]. The adenomatous histotype can be tubular (more than 80%), villous (5–15%), and tubular/villous (5–15%). Hyperplastic polyps are common and carry a small risk of evolving into cancer [8]. Although the different types of polyps may be diffused in the large bowel, adenomatous and hyperplastic polyps are mainly located in the distal colon [14,15,16] and sessile serrated polyps are frequently found in the proximal colon [17,18,19,20]. Owing to the malignant potential of tubular/villous adenomas, patients diagnosed with adenomatous polyposis, i.e., the constitutive development of multiple colorectal adenomas, are at an increased risk of developing CRC. Most studies investigating the carcinogenesis of *CRA* have focused on villous and familial adenomatous polyps, which have the highest rates of carcinogenesis [21,22], whereas few studies have investigated sporadic tubular adenomas, which has the highest clinical incidence [23,24]. Polyps serve as direct precursors to colorectal cancer in families with a history of polyposis syndrome, as well as in the general population. Genetic events, such as the gain or loss of function of molecules essential for intestinal cell homeostasis, may lead to the development of polyps [25].

The Wnt/β-catenin signaling deregulation is an early event in the onset of colorectal adenoma [26]. Its upregulation is mainly due to the altered functions of the adenomatous polyposis coli (APC) protein, which reduces the differentiation of intestinal epithelial cells (IECs), leading to the onset of adenoma and CRC progression. Furthermore, the mutations and LOH of *APC* alter the quantitative regulation of the β-catenin protein, which accumulates in the nucleus, favoring the activity of transcription factors for cell proliferation gene expression and reducing differentiation [27]. Familial adenomatous polyposis (FAP) accounts for less than 1% of CRC cases. It is an inherited CRC syndrome caused by a germline mutation in the *APC* gene, inherited in an autosomal dominant pattern. Around 70% of patients with FAP have a family history of colorectal polyps and cancer. FAP is characterized by the growth of many tens to thousands of adenomas in the rectum and colon during the second and third decade of life. APC is essential for IEC homeostasis and its inactivation facilitates tumorigenesis. Indeed, *APC* somatic truncation mutations are observed in more than 90% of human colon cancers [28,29]. *Wnt* ligands may activate the canonical (β-catenin-dependent) and the non-canonical (β-catenin-independent) pathways. They work in concert to maintain the renewal, defense, and metabolic homeostasis of the colon epithelia [30].

Most of the cellular β-catenin is confined to the adherens junctions on the plasma membrane. Cytosolic β-catenin associates in a complex with APC and axis inhibition protein 1 (AXIN1) proteins, which mediate the N-terminal phosphorylation of β-catenin. This event conducts the ubiquitination of β-catenin by the beta-transducin repeat containing E3 ubiquitin protein ligase (β-TRCP) following proteasomal degradation. When Wnt ligands bind to the Frizzled receptors, Dvl/Dsh is phosphorylated and, in turn, recruits AXIN1 and glycogen synthase kinase-3 beta (GSK3β) adjacent to the plasma membrane, thus preventing the building of the degradation complex. Consequently, unphosphorylated β-catenin eludes recognition by β-TRCP and moves into the nucleus. There, it binds to the T-cell factor (TCF) and lymphoid enhancer-binding protein family (LEF) transcription factors.

The activated β-catenin/TCF/LEF complex triggers gene transcription that regulates cell proliferation and survival. In normal cells, two LEF1 isoforms regulate *Wnt*-dependent pathways as apoptosis, motility, and gene transcription, and its expression in human colon tissue gradually increased from a normal colon, low-grade adenoma, high-grade adenoma, to adenocarcinoma [31]. Then, β-catenin accumulates in the cytoplasm and in the nucleus [32].

The B-cell CLL/lymphoma 9 (BCL9) protein is a novel co-factor of canonical Wnt/β-catenin signaling [33,34,35]. It forms a complex with β-catenin-LEF/TCF to activate the transcription of Wnt target genes, after the hyper-activation of canonical Wnt signaling [36]. In CRC tissues, *Wnt3* is highly expressed to sustain autocrine Wnt activity and CRC progression by EMT and is indicative of advanced stages with poor prognoses [37]. Inhibiting *Wnt3* secretion inhibits colon cancer cells proliferation [38]. The *Wnt3a* expression was also increased and associated with EMT, which is indicative of advanced stages with poor prognoses [39]. Moreover, *Wnt3a* was overexpressed in CRC primary tissues than in metastatic areas, suggesting that *Wnt3a* was expressed early in cancer rather than appearing as it progressed. A more recent study discovered that *Wnt3a* inhibits the ability of human colon myofibroblasts to proliferate and migrate [40]. Thus, various CRC subgroups have distinct molecular and cellular properties contributing to *Wnt3a*’s context-dependent nature.

*Wnt5a* is a potent non-canonical Wnt ligand that strongly antagonizes and, ultimately, suppresses the functions of canonical Wnt ligands [41]. Recent research shows that the *Wnt5a* non-canonical ligand can act as both a tumor suppressor and an oncogenic agent, promoting and inhibiting tumor processes through canonical and non-canonical Wnt signaling [42,43]. The exact role of *Wnt5a* in CRC is contradictory [44].

As described above, Wnt/β-catenin signaling is well-known in human CRC, but less studied in the adenoma formation in the early stages of colon tumorigenesis. Based on what is reported in the current literature, there are no studies analyzing these aspects on human adenomas. The main aim of this study was to try to fill the gap that exists in the literature on molecular alterations during adenoma formation and in the very early stages of colorectal carcinogenesis. Only the early inactivation of the *APC* gene is known. It is the most studied gene of the Wnt pathway and there are many mutational analysis studies of the *APC* gene but few on gene and protein expression. The first aim of this study was to study the *APC* gene, and then to analyze less studied Wnt pathway genes, such as *Wnt3a*, *Wnt5a*, *LEF1*, and *BCL9*. To complete the study, we analyzed *β-catenin*, whose role in colorectal carcinogenesis is known, but little is known about its role in adenoma formation. Finally, we investigated the relationships among these key elements of Wnt/β-catenin signaling.

The study also aimed to verify whether there were molecular alterations in the tissues surrounding and adjacent to the tumor, to provide clinical insights for disease management.

The analyses of the gene and protein expression were conducted on pathological samples (polyps and related adjacent mucosa) derived from patients both with sporadic adenomas and suffering from FAP.

## 2. Materials and Methods

### 2.1. Tissues

Pathological and control tissues were recruited from patients with sporadic adenomas undergoing endoscopic biopsy at the Digestive Endoscopy and Gastroenterology Unit, “Santissima. Annunziata” Hospital of Chieti, while those from patients belonging to families with FAP were recruited at the Surgery Unit, Careggi University Hospital in Florence. The collection of each pathological sample was accompanied by the normal-appearing tissue sample obtained from areas that were at least 5 cm away from the margins of the primary lesion. Finally, normal colonic mucosal samples were collected from healthy donors with age ≥18 years without inflammatory bowel disease and personal or family history of cancer, who had undergone follow-up colonoscopy at the Digestive Endoscopy and Gastroenterology Unit, “Santissima. Annunziata” Hospital of Chieti (UOD of Digestive Endoscopy and Gastroenterology). After the surgical removal, the tissue fragments were stored in RNAlater™ solution (Thermo Fisher Scientific, Waltham, MA, USA) to stabilize the RNA and to preserve proteins, and subsequently stored at −80 °C. The study protocol was approved by the local Ethics Committee and each participant tissue donor provided written informed consent. The study was conducted according to the Declaration of Helsinki and approval was granted from the Institutional Review Board (Prot. Id. RICH1KHE). Adenoma tissues were classified according to conventional histopathological criteria, as defined by World Health Organization (WHO). Patient characteristics and polyp histotype are shown in Table 1.

Tissues were selected based on RNA availability. Samples with insufficient quantity of target in the cDNA template or protein were not included in the gene and protein expression analyses. The study included 68 colonic samples; 58 biopsies (33 polyps and 25 adjacent mucosa) belonged to 41 patients, of which 17 were affected by FAP, and ten normal colonic mucosal samples were collected from 10 healthy donors.

### 2.2. Real-Time Quantitative PCR Analysis (qRT-PCR)

Total RNA was separated from colon tissues homogenized in liquid nitrogen with a mortar and pestle, using TRIzol^®^ Reagent (Applied Biosystems, Thermo Fisher Scientific, Waltham, MA, USA) following the manufacturer’s instructions at RNase-free atmosphere. The RNA samples were assessed for purity and quantified using a Nanodrop 1000 Spectrophotometer (Applied Biosystems, Thermo Fisher Scientific, Waltham, MA, USA). Complementary DNA (cDNA) were synthesized as previously described [46]. The mRNA levels were evaluated by SYBR Green and TaqMan assay by quantitative real-time PCR (qRT-PCR) analysis using StepOne™ 2.0 (Applied Biosystems, Thermo Fisher Scientific, Waltham, MA, USA). Data were analyzed by the comparative Ct method and graphically represented as 2^−ΔΔCt^ ± SD. In accordance with this method, the mRNA amounts of the target genes were normalized by the ratio on the median value of the endogenous housekeeping gene (GUSB). Primer sequences are available in Catalano et al., 2021 [46]. The cycling conditions were performed as follows: 10 min at 95 °C and 40 cycles of 15 s at 95 °C, followed by 1 min at 60 °C, and final elongation of 15 s at 95 °C. All data were validated in a second analysis. The sequences of paired oligonucleotides were as follows:

- 5′-GCTTGATAGCTACAAATGAGGACC-3′ and 5′-CCACAAAGTTCCACATGC-3′ for APC; RefSeq: [NM_000038];
- 5′-CATGAACCGCCACAACAAC-3′ and 5′-TGGCACTTGCACTTGAGGT-3′ for *WNT-3a*; RefSeq: [NM_033131];
- 5′-CTCATGAACCTGCACAACAACG-3′ and 5′-CCAGCATGTCTTCAGGCTACAT-3′ for *WNT-5a*; RefSeq: [NM_03392];
- 5′-CCAACTTGCCATCAATGAATAA-3′ and 5′-GGCATCTGATTGGAGTGAGAA-3′ for BCL-9; RefSeq: [NM_004326];
- 5′-GAC GAG ATG ATC CCC TTC AA-3′ and 5′-AGG GCT CCT GAG AGG TTT GT-3′ for LEF-1; RefSeq: [NM_016269];
- 5′-AGCCAGTTCCTCATCAATGG-3′ and 5′-GGTAGTGGCTGGTACGGAAA-3′ for GUSB; RefSeq: [NM_000181].

### 2.3. Western Blotting

Total proteins were isolated from pathological and control tissues homogenized in liquid nitrogen with a mortar and pestle, using RIPA lysis buffer (Cell Signaling Technology, Beverly, MA, USA). Proteins were quantified by Bradford Assay (Thermo Fisher Scientific, Waltham, CA, USA) and the protein lysates were subjected to electrophoresis, followed by immunoblotting. Then, 30 µg of total proteins was incubated with SDS-PAGE sample buffer (125 mM Tris-HCl, pH 6.8, 4% SDS, 20% glycerol, 10% beta-mercaptoethanol, and 0.004% bromophenol blue) at 100 °C for 5 min. Tris-glycine SDS running buffer (25 mM Tris, 250 mM glycine, 0.1% SDS) was used for electrophoresis. Proteins were separated for 60′ at 100 V in an 8% Tris/glyicine acrylamide gel (Mini PROTEAN electrophoresis cell). After electrophoresis, proteins were transferred onto the Immobilon-P PVDF membrane at 80 mA for 1 h by using Tris-glycine SDS (48 mM Tris, 39 mM glycine, 0.037% SDS) transfer buffer with 20% methanol in a Mini Trans-Blot Electrophoretic Transfer Cell. The blotted PVDF membranes were directly blocked with 5% bovine serum albumin (BSA) in Tris-buffered saline with 0.05% Tween-20 (TBST) for 1 h and incubated overnight 4 °C with primary antibodies diluted in TBST with 5% BSA. Primary antibodies were Active-β-Catenin (1:1000) (Cell Signaling Technology), APC (1:1000) (Merck-Millipore, Burlington, MA, USA), and β-actin (1:8000) (Sigma-Aldrich, St. Louis, MI, USA), used as a protein loading control. The blotted membranes were washed thoroughly with TBST before incubation with diluted (1:10,000) HRP-conjugated anti-rabbit or anti-mouse (Bethyl Laboratories, Montgomery, TX, USA). The immune complexes were visualized using the ECL western blot detection system (EuroClone) by using AllianceLD2 hardware (UVItec Limited, Cambridge, UK).

### 2.4. Statistical Analysis

All measurements were made after three independent experiments were conducted under the same experimental condition. A mean value of all experiments plus standard deviation *(SD)* was shown for qRT-PCR data, while, for western blotting, a representative value was shown. Statistical analyses were performed using SPSS software 29.0. Kruskal–Wallis non-parametric test for independent samples was used to compare the expression of the five genes in polyps, adjacent mucosa, and normal tissue for each gene separately. Spearman’s ρ correlation coefficients were evaluated between the five genes. *p*-value was considered as significant if <0.05. Microsoft Excel version 16.66.1 was used to draw a scatter plot and linear trend line. Descriptive statistics were carried out using GraphPad Prism version 9.0 (GraphPad Software Inc., La Jolla, CA, USA).

## 3. Results

We analyzed the expression of five genes of the Wnt/β-catenin family in all available tissues, grouping them into three categories: colorectal adenomas, adjacent mucosa, and normal tissues. To evaluate any alterations in the protein expression of APC and β-catenin in the mucosa adjacent to the adenoma transition, we performed protein expression in the cases in which the matched adenoma and adjacent mucosal tissues were available.

### 3.1. Gene Expression of APC, Wnt3a, Wnt5a, BCL9, and LEF1 in FAP and Tubular–Villous Adenomas

The gene expression of the five Wnt/β-catenin signaling genes (*APC*, *BCL9*, *LEF1*, *Wnt3a*, and *Wnt5a*) was conducted on 52 colonic samples; 42 biopsies (25 polyps and 17 adjacent mucosa) belonged to 33 patients, of which 15 were affected by FAP, and 10 were normal colonic mucosal samples from 10 healthy donors.

**Figure 1:**
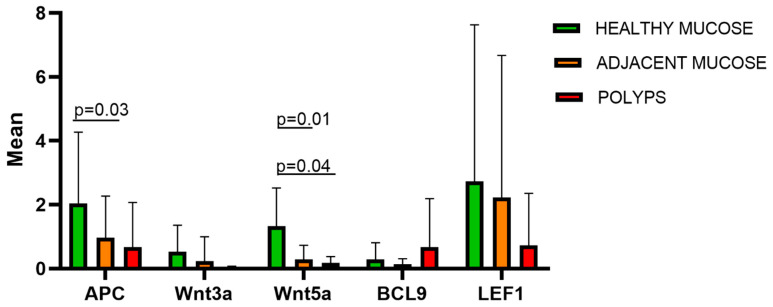
The figure shows the mean expression for five Wnt/β-catenin pathway genes (*APC*, *Wnt3a*, *Wnt5a*, *BCL9*, and *LEF1*) in healthy colorectal mucosa (n.10), adjacent mucosa (n.17), and polyps (n.25). Significant differences were detected in *APC* and *Wnt5a* expressions in polyps compared to normal tissue (*p* = 0.03), and, for *Wnt5a* expression, also in polyps compared to normal tissue (*p* = 0.04). *APC:* adenomatous polyposis coli; *Wnt3a*: *Wnt* family member 3a; *Wnt5a*: *Wnt* family member 5a; *BCL9*: B-cell CLL/lymphoma 9; *LEF1*: lymphoid enhancer-binding factor 1.

The expressions of *APC*, *Wnt3a*, *Wnt5a*, *BCL9*, and *LEF1* in the polyps, adjacent mucosa, and normal tissue were compared by the Kruskal–Wallis non-parametric test for independent samples. Significant results were found for *APC* (*p* = 0.03) and *Wnt5a* (*p* = 0.01)**.** A post hoc test showed a significant difference in *APC* expression for polyps compared to healthy mucosa (*p* = 0.03) (Figure 1), and in *Wnt5a* expression for healthy mucosa compared to both polyps (*p* = 0.04) and adjacent mucosa (*p* = 0.01).

**Figure 2:**
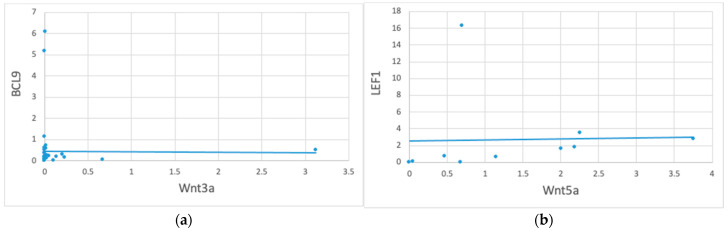
Correlation between *Wnt3a* vs. *BCL9* and *Wnt5a* vs. *LEF1* gene expression in 52 colon tissue samples. Correlation is significant at the 0.05 level (2-tailed). The figure represents a scatter plot and linear trend line of the expression values between *Wnt3a* vs. *BCL9* in polyps and adjacent mucosa (n. 42) (panel (**a**)) and of *Wnt5a* vs. *LEF1* in normal colonic mucosa (n. 10) (panel (**b**)).

Furthermore, the Spearman’s ρ analysis revealed significant correlations between *Wnt3a* and *BCL9* (ρ = 0.34, *p* = 0.03) when the polyps and adjacent mucosa were considered in the same category, and between *Wnt5a* and *LEF1* (ρ = 0.70, *p* = 0.03) for healthy tissues. A scatter plot and linear trend line are shown in Figure 2.

**Figure 3:**
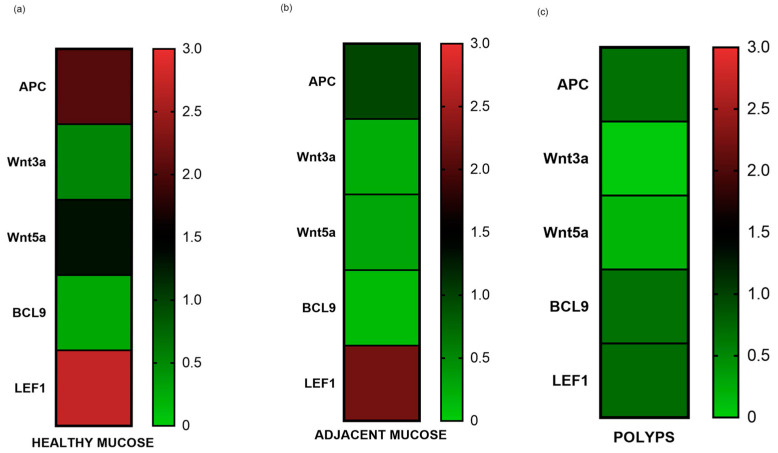
The figure represents a heat map of the gene expression means of *APC*, *Wnt3a*, *Wnt5a*, *BCL9*, and *LEF1* in healthy mucosa (**a**), adjacent mucosa (**b**), and polyps (**c**). It is shown how gene expression tends to reduce from healthy to pathological tissue.

The gene expression results were graphically represented using a heat map. *APC*, *Wnt3a*, *Wnt5a*, *BCL9*, and *LEF1* tend to reduce from healthy to pathological tissue (Figure 3). *APC*, *Wnt5a*, and *LEF1* are the most expressed genes in healthy mucosa (Figure 3).

### 3.2. Protein Expression Assay by Western Blotting of APC and β-catenin

**Figure 4:**
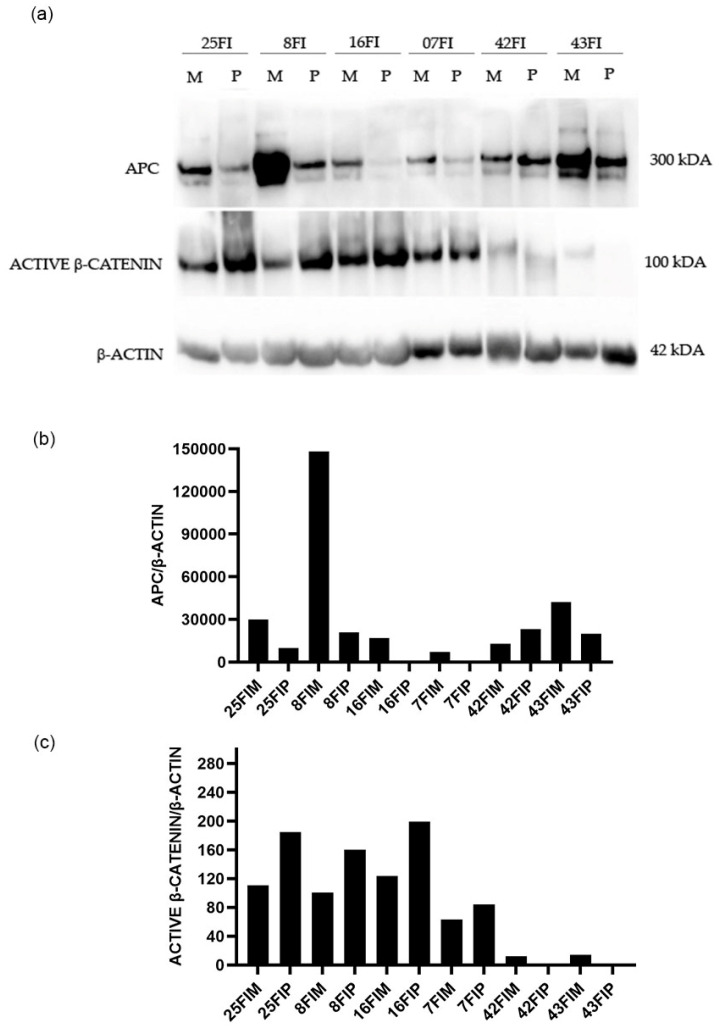
Western blotting analysis in familial adenomas determining the protein expression levels of APC and β-catenin in polyp (P) vs. adjacent mucosa (M). Data shown are representative of three independent experiments. The expression levels of panel (**a**) were determined by densitometric analysis (panel (**b**,**c**)) and calculated in relation to the β-actin level. kD: kilodalton as protein molecular weight unit.

**Figure 5:**
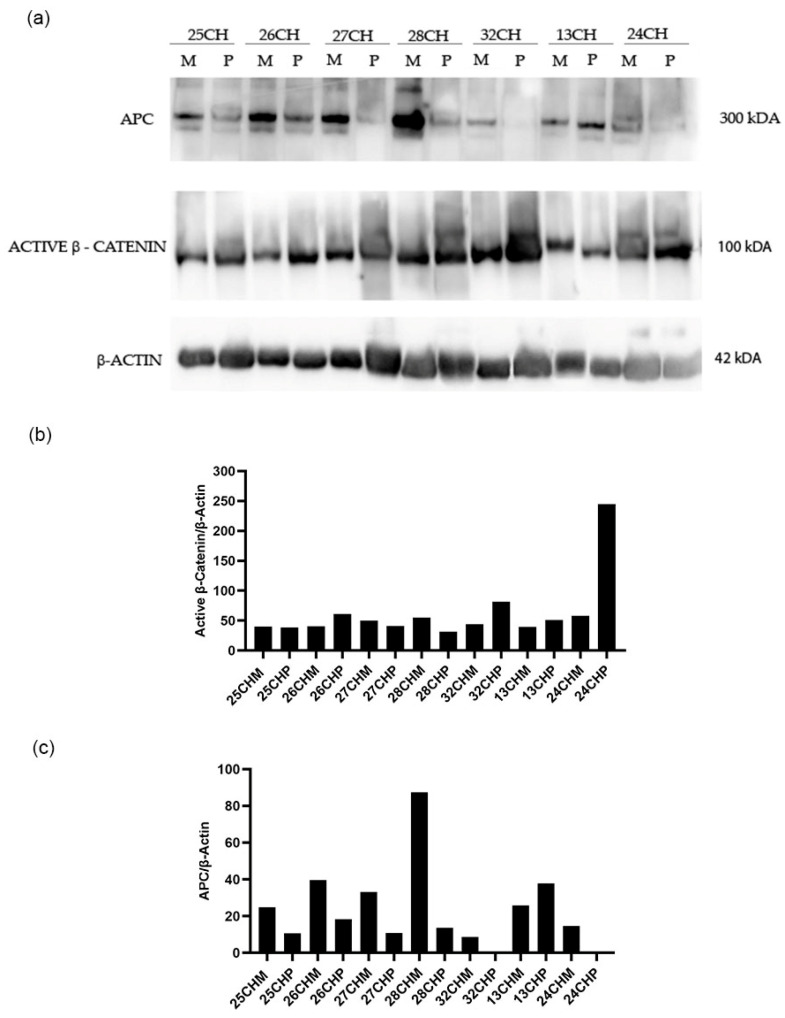
Western blotting analysis in sporadic adenomas determining the protein expression levels of APC and β-catenin in polyp (P) vs. adjacent mucosa (M). Data shown are representative of three independent experiments. The expression levels of panel (**a**) were determined by densitometric analysis (panel (**b**,**c**)) and calculated in relation to the β-actin level. kD: kilodalton as protein molecular weight unit.

A 50% reduction of APC gene expression in the adjacent mucosa compared to the healthy mucosa, and a more accentuated reduction in polyps, have been detected (Figure 1). Therefore, we wanted to investigate the levels of APC and β-catenin proteins in still available tissues. Then, a western blot analysis of matched polyp-adjacent mucosal tissues was performed. The matched adenoma and adjacent mucosal tissues were available for six FAP patients and for seven patients with sporadic adenomas. The analyses revealed the expression of the full-length APC protein (300 kDa band) in all matched adenoma-adjacent mucosa samples (Figure 4a and Figure 5a). A decrease in APC expression is visible in all the adenomas, both familial and sporadic, compared to adjacent mucosa. As we expected, as regards the β-catenin expression, its active form appeared to be increased in all the familial and sporadic adenomas compared to adjacent mucosa (Figure 4a and Figure 5a). Sample 42FI-P does not show a decrease in APC expression, and, intriguingly, it shows a reduced active β-catenin expression, also visible in the matched adjacent mucosa. Moreover, for sample 43FI, a reduced expression of the active β-catenin was noted, apparently not associated with APC modulation. The relative expression quantification of the selected proteins (APC and active β-catenin) detected by WB in the available matched adenoma and adjacent mucosal tissues are shown in Figure 4b,c and Figure 5b,c.

## 4. Discussion

The current literature lacks significant Wnt/β-catenin/APC gene expression studies on adenomas. Our study is the first to analyze the modulation of the Wnt/β-catenin pathway in early carcinogenesis. Understanding the significance of benign pre-cancerous lesions is widely recognized; yet, studying them is complicated in terms of sampling, mainly due to the difficulty in obtaining biopsies and their small size. The long period required for polyps and cancer to develop, as well as the tendency of early-stage cancers to be asymptomatic in many individuals, allows a window of opportunity for polypectomy and cancer prevention, as well as for early diagnosis and highly effective drug administration for early-stage cancers. Blood-based tests could detect genetic changes associated with polyps released into circulation. Promoting the uptake and completion of follow-up testing and treatment holds significant potential to save lives. Therefore, understanding the progression from colorectal adenomas to colorectal carcinomas is essential for the development of new therapeutic strategies and improving clinical outcomes.

Tumor and adjacent mucosa biopsies are the definitive diagnostic procedures for a histological evaluation. The adjacent mucosa, also known as “apparently healthy” mucosa, being closely associated with polyps in the tumor microenvironment (TME), may have already been impacted molecularly [47]. Our data support this hypothesis.

Initially, the study was designed to analyze any molecular differences between sporadic adenomas and FAP, but the sample size did not allow this. It is already known that *APC* is inactivated in FAP polyps. This study focused on evaluating the APC gene and protein expression not only on pathological FAP tissue and tubular–villous adenomas, but also on apparently normal endoscopic mucosal tissue sampled at the 5 cm proximal. Very interestingly, a lower APC expression was detected in the adjacent mucosa compared to the normal one, but, at the same time, it is the most expressed gene in the healthy mucosa together with *LEF1* and *Wnt5a*. This is explained because it is the regulator of the Wnt pathway in a colonic tissue adjacent to the tumor but in which it still retains its functions.

APC has a functional role in the canonical (β-catenin-dependent) Wnt signaling pathways [48]. Most cases of CRCs are initiated by *APC*-inactivating mutations, leading to the constitutive activation of the Wnt/β-catenin signaling pathway. Most of the literature has extensively assessed the role of APC somatic and germline mutations in familial as well as sporadic forms of CRC.

It is known that *APC* is inactivated in FAP polyps. Our results showed a reduction in APC expression both in FAP and sporadic polyps. Additionally, a lower APC expression was detected in the adjacent mucosa.

Moreover, APC can inhibit the initiation and development of colorectal tumor, independently of canonical Wnt signaling. APC assists in chromosome segregation, establishes cellular polarity and migration, and represses DNA replication [27]. *APC* mutations contribute in early adenoma creation leading to chromosomal instability by triggering spindle abnormalities and the deregulation of microtubules/the actin cytoskeleton. Moreover, *APC* mutations increase cell migration by reducing cell adhesion via the deregulation of β-catenin and E-cadherin distributions among the cytoplasm and the cell membrane [49]. Xu et al. demonstrated that the overexpression of the erythropoietin-producing hepatocyte (EphB6), a member of the tyrosine kinase family, along with *APC* gene mutations, increases proliferation, migration, and invasion in the colon epithelial cell line, IMCE, supporting the role of *APC* mutations in promoting tumorigenesis in CRC [50]. In our study, the APC gene was less expressed in the colon tumor compared to the adjacent colonic mucosa and to the mucosa of healthy controls. Moreover, the APC protein was less expressed in the colon tumor compared to the adjacent colonic mucosa. All results confirmed the literature data on carcinomas.

LEF1 is a downstream mediator and nuclear effector of the Wnt/β-catenin signaling pathway [51]. In normal cells, two LEF1 isoforms are in regulation of *Wnt*-dependent pathways as apoptosis, motility, and gene transcription. The role of LEF1 is controversial, usually detected and upregulated in most colonic carcinomas, enhancing the progression [52]. Nevertheless, little is known about the expression of LEF1 in early carcinogenesis. LEF1 is frequently highly expressed in tumor development, potentially driving cancer proliferation and spread [53]. Reducing LEF1 in glioblastoma multiforme cells restricts their ability to invade, migrate, and proliferate, as well as the self-renewal capability of stem-like cells [54]. Myc controls the expression of LEF1 to activate the Wnt pathway in colon cancer [55]. The enzyme lysine-specific demethylase 1 (LSD1) also promotes bladder cancer progression by enriching LEF1 expression and improving EMT [56]. Although the LEF1 expression may play a role in cancer development [57,58,59], insufficient evidence supports its involvement in malignant phenotype changes. The molecular mechanisms regulating LEF1 in the advancement of colonic adenocarcinoma are currently unknown. However, a study by Xiao et al. in 2021 [52] keeps LEF1 as a potential therapeutic target for colonic adenocarcinoma, suggesting that it enhances the motility of cancer cells by reshaping the lamellipodia/filopodia and the polymerization of F-actin/β-tubulin. The Xiao et al., 2021 study findings support LEF1 as a potentiator and potential therapeutic target for colonic adenocarcinoma. LEF1 increases colonic adenocarcinoma cells’ motility by remodeling the lamellipodia/filopodia and the polymerization of F-actin/β-tubulin. LEF1 maintains the viability and growth of colonic adenocarcinoma cells through improving proliferation and Lamin B1 expression, and reducing apoptosis. Furthermore, LEF1 is nearly linked with EMT. However, an in vivo study published in Science [60] showed that *LEF1* restricts ectopic crypt formation and tumor cell growth in colon adenomas from APC-deficient mice. The loss of *Lef1* markedly increased tumor initiation and tumor cell proliferation, reduced the expression of several Wnt antagonists, and increased the *Myc* proto-oncogene expression and the formation of ectopic crypts in *Apc*-mutant adenomas. These results uncover a previously unknown negative feedback mechanism in CRC, in which the ectopic *Lef1* expression suppresses intestinal tumorigenesis by restricting adenoma cell dedifferentiation to a crypt-progenitor phenotype and by reducing the formation of cancer stem cell niches. The recent literature data therefore demonstrate how the controversy on the function of *LEF1* in colorectal tumorigenesis is still open.

There are many articles in the literature that underline the role of *LEF1* as a transcription factor and, therefore, its increased presence in cancer. But there are others, such as the one published in Science [60] in which its loss increased cell growth. In our study, *LEF1* is highly expressed in the adjacent mucosa and decreased in the polyp, supporting the recent hypothesis of LEF1’s role as a tumor suppressor, already in adenomas, but also its essential role in stem cell maintenance during crypt formation and organ development.

BCL9 is considered a key developmental regulator and a well-established oncogenic driver in multiple cancer types, mainly through potentiating the Wnt/β-catenin signaling. BCL9 is recognized as a crucial part of the nuclear β-catenin complexes [61]. It serves as an adapter protein that provides binding sites for the Wnt signaling transcriptional system [62]. BCL9 functions as an oncoprotein by supporting cancer progression primarily through maintaining cancer cell division [63], promoting proliferation and migration, inhibiting apoptosis [64], remodeling the tumor microenvironment and immune system, and regulating the chromosomal instability and karyotype for tumor evolution [65].

*Wnt3a* is a ligand that triggers the canonical Wnt pathway. In glioblastoma [66], breast and prostate tumors [67,68], and malignant mesothelioma [69], *Wnt3a* stimulates the development of cancer. Furthermore, research has indicated that *Wnt3a* is a tumor suppressor [70]. Marit et al. noted that *Wnt3a* suppresses the growth of multiple B-acute lymphoblastic leukemia cell lines [71]. Qi L. et al. [39] examined the expression of *Wnt3a* in numerous colon cancer tissue samples to investigate its impact on colon cancer progression. They found a strong connection between the *Wnt3a* expression and histological differentiation, clinical stages, metastasis, and recurrence. The data obtained indicate that the upstream component of the Wnt signaling pathway could have a significant impact on the advancement of colon cancer, which is consistent with a recent investigation on colorectal cancer that found high levels of *Wnt3a* expression in both primary and metastatic locations, and its strong correlation with the presence of the metastasis-related protein matrix metalloproteinase (MMP)-9 [72]. In our study, the correlation between BCL9 and *Wnt3a* both in polyps and in the adjacent mucosa confirms two aspects: the first is that the canonical Wnt pathway is already activated in the adjacent mucosa; the second is that it is also activated in early carcinogenesis. This correlation concerns the adenoma formation, regardless of the clinical characteristics of the patient, FAP or sporadic. Finally, our results confirm *Wnt3a* as a tumor suppressor.

The precise role of *Wnt5a* still needs to be supported by conclusive evidence. Specific research indicates that *Wnt5a* is a tumor suppressor, while others propose the contrary. In the progression of colorectal cancer, *Wnt5a* demonstrates diverse roles across various signal transduction pathways [73]. The mRNA of *Wnt5a* is present in most healthy tissues, including the colon. However, its expression significantly increases during the transition from normal tissue to carcinomas [74]. On the other hand, the presence of the *Wnt5a* protein appears to decrease, as it is commonly deactivated in colorectal cancer (CRC) through tumor-specific methylation. This makes it a potential biomarker [75]. *Wnt5a* is believed to function as a tumor suppressor in CRC by inhibiting the Wnt/β-catenin signaling pathway [75].

Traditionally, *Wnt5a* has been considered to be the non-canonical Wnt ligand and triggers Ca2+-dependent effectors and other non-canonical pathways through small Rho-GTPases and c-Jun-NH2-kinase [76]. However, its role in the progression of CRC is intricate and appears to be paradoxical. Multiple studies have demonstrated that *Wnt5a* is silenced in the majority of CRC cell lines and samples because of the frequent methylation in its promoter region [77,78]. The expression of *Wnt5a* showed a negative association with the level of tumor differentiation and the aggressive behavior [77,79].

Meanwhile, the methylation of the *Wnt5a* promoter was closely linked to the microsatellite instability status of colorectal cancer (CRC) patients, and various histone modifications of *Wnt5a* participated in its suppression and could potentially encourage the spread of colon cancer, suggesting that epigenetic processes might improve the *Wnt5a*-mediated signaling in CRC [80,81]. Conversely, other research indicated that *Wnt5a* was consistently overexpressed in intestinal polyps and tumor samples, and elevated *Wnt5a* levels were associated with early recurrence or metastasis in colon cancer patients [82,83]. *Wnt5a* was also found to facilitate the movement of CRC cells by activating Fzd7-driven non-canonical Wnt signaling and enhance CRC cells’ stemness by activating canonical Wnt signaling [84,85].

Our study showed *Wnt5a* expressed in healthy mucosa. The correlation between *Wnt5a* and LEF1 detected in healthy tissues could indicate that the Wnt non-canonical pathway is active in normal colonic tissue. Furthermore, these results could also denote a possible inflammatory state of donors undergoing a screening colonoscopy, as both *Wnt5a* and LEF-1 are linked to the inflammatory state. Aberrant Wnt signaling is linked to defects in the chronic inflammatory response. Indeed, in the still normal colonic mucosa, various inflammatory mediators can actively contribute to the creation of a TME favorable to cell transformation, survival, and proliferation [86]. The mutual interaction of epithelial cells within the TME influences the stages of tumorigenesis driven, to a large extent, by inflammation. This may be an attractive therapeutic target to control inflammation in the colonic mucosa [87]. Furthermore, IEC can drive the plasticity of stromal cells in the TME under the influence of extrinsic factors, such as diet, and the microbiota composition contributing to inflammation and tumorigenesis in CRC [86,88]. However, understanding the interactions between Wnt signaling and inflammatory/immune responses in tumor onset remains a necessary goal for both prevention and therapy, given that the majority of CRCs appear to be immunologically “cold” tumors unresponsive to therapies with immune checkpoint inhibitors [89].

The observed correlations between *Wnt3a* and *BCL9* gene expressions and between *Wnt5a* and *LEF1* could provide biological significance such as the activation of the canonical *Wnt* pathway in pathological tissues and the non-canonical pathway in healthy tissues, although they require further studies.

*APC* and β-catenin were downregulated and upregulated, respectively, already in the adenoma formation. As regards *Wnt3a*, *Wnt5a*, *BCL9*, and *LEF*, the study underlined a modulation of these genes in pathological tissue compared to normal tissue.

The study showed that the mucosa adjacent to adenoma is already altered at a molecular level. This result provides a new clinical insight: verifying the nature of the colorectal mucosa during follow-up by analyzing markers such as APC and β-catenin.

These findings provide information on the possible progression towards carcinoma of the residual mucosa, already altered at a molecular level. The investigation would take place during follow-up and, therefore, would not add further medicalization to the patient. To contain costs, it could initially be aimed at FAP patients, who have a greater risk of recurrence of adenomas, rather than sporadic patients.

### Limitations

This is an observational study, and additional investigations are needed to facilitate the understanding of the potential clinical implications of the results. *APC* has been validated with two independent techniques, qRT-PCR and WB. It has not been possible to validate the results from the analyses of *Wnt3a*, *Wnt5a*, *BCL9*, and *LEF1* with another independent technique, due to the small size of the biopsies, which, therefore, constituted a limiting factor. The results regarding these four genes should be confirmed on a larger sample, before hypothesizing about their use as markers in the clinic. There is very little scientific literature concerning both carcinomas and adenomas, and even validation using datasets such as The Human Protein Atlas (https://www.proteinatlas.org, accessed on 29 July 2024) has not provided us with the possibility of a comparison.

## 5. Conclusions

This is the first study analyzing the gene expression of *APC*, *Wnt3A*, *Wnt5A*, *BCL9*, and *LEF1* in the colon polyps vs. adjacent mucosa and vs. normal mucosa from control individuals. These findings of altered expression levels of Wnt genes in apparently normal adjacent mucosa from patients with familial and sporadic colon polyps underline an interplay between the tumor and the surrounding colonic epithelium. This may aid in identifying patients at risk of developing cancer.

In conclusion, our study aims to enhance the comprehension of the pathogenesis in colorectal adenomas (CRAs) and to propose the utilization of APC and β-catenin as markers in clinical settings. Identifying crucial genes, investigating their potential role in the pathogenesis of colorectal adenomas, and developing gene-targeted medications are pressing clinical and scientific issues that need to be addressed.

## Figures and Tables

**Table 1:** Clinical and histopathological characteristics of 41 patients with FAP and sporadic adenomas analyzed.

	**Patients with FAP Polyps**					
**Case**	**Age**	**Sex**	**Phenotype**	**Site and Size of Polyps**	**Dysplasia** **(L or H)**	***n*** **. of Polyps**
5FI	25	F	Adenomatous	Diffuse or “carpet”, <1 cm	HGD	1060
6FI ^a,e^	58	M	Adenomatous	Diffuse	HGD	25
7FI ^a,e^	28	F	Adenomatous	Diffuse	HGD	375
8FI ^b,e^	18	F	Adenomatous (Tubular–villous)	Diffuse	HGD	415
9FI ^c,e^	15	F	Adenomatous	Diffuse	LGD	375
16FI	n.a	F	Adenomatous	Diffuse	LGD	n.a
25FI	n.a.	M	Adenomatous	Diffuse	LGD	n.a.
26FI	46	F	Adenomatous	Diffuse	LGD	835
31FI		M	Adenomatous	Diffuse		
33FI	49	F	Adenomatous	Diffuse	LGD	97
35FI ^c,e^	31	M	Adenomatous	Diffuse	LGD	550
36FI	42	F	Adenomatous	Diffuse	LGD	250
39FI	42	M	Adenomatous	Diffuse	LGD	430
40FI ^a,d,e^	61	F	Adenomatous	Diffuse	HGD	730
41FI	49	M	Adenomatous	Diffuse	LGD	1025
42FI	42	F	Adenomatous and amartomatous	Diffuse	LGD	210
43FI	36	M	Adenomatous	Diffuse	LGD?	n.a
	**Patients with Sporadic Polyps**					
**Case**	**Age**	**Sex**	**Phenotype**	**Site and Size of Polyps**	**Dysplasia** **(L or H)**	**Morphology**
1CH	50	M	Hyperplastic	Sigma, 6 mm	LGD	Spl
2CH	67	M	Tubular	Sigma, 10 mm	LGD	Spl
3CH	49	M	Hyperplastic	Sigma, 4 mm	LGD	Spl
9CH	47	M	Tubular–villous	Retto, 15 mm	LGD	Ppl
11CH	57	F	Hyperplastic–adenomatous	Descending, 4 mm	LGD	Spl
13CH	83	F	Tubular	Descending, 15 mm	LGD	Spl
15CH	37	F	Villous	Sigma, 50 mm	HGD	Ppl
16CH	60	M	Tubular	Cecum, 15 mm	LGD	Ppl
17CH	66	M	Tubular–villous	Sigma, 15 mm	LGD	Ppl
18CH	64	M	Tubular–villous	Descending, 8 mm	LGD	Spl
21CH	78	M	Tubular–villous	Sigma, 10 mm	LGD	Spl
22CH	67	M	Tubular–villous	Rectum, 10 mm	LGD	Spl
23CH	68	F	Tubular–villous	Sigma, 10 mm	LGD	Ppl
24CH	59	M	Tubular–villous	Ascending	LGD	Ppl
25CH	77	M	Tubular–villous	Descending	HGD	Ppl
26CH	69	M	Tubular	Splenic flexure, 10 mm	LGD	Spl
27CH	61	F	Tubular–villous	Sigma, 15 mm	LGD	Ppl
28CH	77	M	Tubular–villous	Hepatic flexure, 5 mm	LGD	Spl
29CH	47	M	Hyperplastic–adenomatous	Descending, 20 mm	Not atypical	Ppl
30CH	53	M	Hyperplastic–adenomatous	Retto-sigma, 7 mm	Not atypical	Spl
31CH	76	M	Tubular	Ascending, 5 mm	LGD	Spl
32CH	51	M	Tubular–villous	Ascending, 45 mm	LGD	Spl
33CH	68	F	Tubular–villous	Colon, 40 mm	LGD	LST-G
34CH	67	M	Tubular	Colon sx, 7 mm	LGD	Ppl

^a^ Presence of rectal cancer; ^b^ APC mutation: c.4666_4665ins(p.Thr1556fs); ^c^ APC mutation: c.2805 C>(p.Tyr935X); ^d^ APCmutation: c.3927_3931del(p.Glu1309_Asp.fsx1312); ^e^ [45]. Ppl: pedunculated polypoid lesion; Spl: sessile polypoid lesion; LST-G: laterally spreading tumor (LST)—granular shape (G); HGD: high-grade dysplasia; LGD: low-grade dysplasia, FAP: familial adenomatous polyposis; FI: case from hospital of Florence; CH: case from hospital of Chieti.

## Data Availability

The original contributions presented in the study are included in the article; further inquiries can be directed to the corresponding author.
